# Formative Evaluation of the Acceptance of HIV Prevention Artificial Intelligence Chatbots By Men Who Have Sex With Men in Malaysia: Focus Group Study

**DOI:** 10.2196/42055

**Published:** 2022-10-06

**Authors:** Mary L Peng, Jeffrey A Wickersham, Frederick L Altice, Roman Shrestha, Iskandar Azwa, Xin Zhou, Mohd Akbar Ab Halim, Wan Mohd Ikhtiaruddin, Vincent Tee, Adeeba Kamarulzaman, Zhao Ni

**Affiliations:** 1 Social and Behavioral Sciences Department, Yale School of Public Health New Haven, CT United States; 2 Section of Infectious Disease, Department of Internal Medicine, Yale School of Medicine New Haven, CT United States; 3 Center for Interdisciplinary Research on AIDS (CIRA) Yale University New Haven, CT United States; 4 Centre of Excellence for Research in AIDS (CERiA), Faculty of Medicine, University of Malaya Kuala Lumpur Malaysia; 5 Division of Epidemiology of Microbial Diseases Yale School of Public Health New Haven, CT United States; 6 Department of Allied Health Sciences, University of Connecticut Storrs, CT United States; 7 Department of Medicine, Infectious Disease Unit, Faculty of Medicine Kuala Lumpur Malaysia; 8 School of Nursing Yale University New Haven, CT United States

**Keywords:** artificial intelligence, chatbot, HIV prevention, implementation science, men who have sex with men, MSM, mobile health design, mHealth design, unified theory of acceptance and use of technology, mobile phone

## Abstract

**Background:**

Mobile technologies are being increasingly developed to support the practice of medicine, nursing, and public health, including HIV testing and prevention. Chatbots using artificial intelligence (AI) are novel mobile health strategies that can promote HIV testing and prevention among men who have sex with men (MSM) in Malaysia, a hard-to-reach population at elevated risk of HIV, yet little is known about the features that are important to this key population.

**Objective:**

The aim of this study was to identify the barriers to and facilitators of Malaysian MSM’s acceptance of an AI chatbot designed to assist in HIV testing and prevention in relation to its perceived benefits, limitations, and preferred features among potential users.

**Methods:**

We conducted 5 structured web-based focus group interviews with 31 MSM in Malaysia between July 2021 and September 2021. The interviews were first recorded, transcribed, coded, and thematically analyzed using NVivo (version 9; QSR International). Subsequently, the unified theory of acceptance and use of technology was used to guide data analysis to map emerging themes related to the barriers to and facilitators of chatbot acceptance onto its 4 domains: performance expectancy, effort expectancy, facilitating conditions, and social influence.

**Results:**

Multiple barriers and facilitators influencing MSM’s acceptance of an AI chatbot were identified for each domain. *Performance expectanc*y (ie, the perceived usefulness of the AI chatbot) was influenced by MSM’s concerns about the AI chatbot’s ability to deliver accurate information, its effectiveness in information dissemination and problem-solving, and its ability to provide emotional support and raise health awareness. Convenience, cost, and technical errors influenced the AI chatbot’s *effort expectancy* (ie, the perceived ease of use). Efficient linkage to health care professionals and HIV self-testing was reported as a *facilitating condition* of MSM’s receptiveness to using an AI chatbot to access HIV testing. Participants stated that *social influence* (ie, sociopolitical climate) factors influencing the acceptance of mobile technology that addressed HIV in Malaysia included privacy concerns, pervasive stigma against homosexuality, and the criminalization of same-sex sexual behaviors. Key design strategies that could enhance MSM’s acceptance of an HIV prevention AI chatbot included an anonymous user setting; embedding the chatbot in MSM-friendly web-based platforms; and providing user-guiding questions and options related to HIV testing, prevention, and treatment.

**Conclusions:**

This study provides important insights into key features and potential implementation strategies central to designing an AI chatbot as a culturally sensitive digital health tool to prevent stigmatized health conditions in vulnerable and systematically marginalized populations. Such features not only are crucial to designing effective user-centered and culturally situated mobile health interventions for MSM in Malaysia but also illuminate the importance of incorporating social stigma considerations into health technology implementation strategies.

## Introduction

### Background

During the past 3 decades, great progress has been made worldwide in HIV prevention, including HIV testing. However, the HIV epidemic in key populations such as men who have sex with men (MSM) continues to grow worldwide in the setting of stigma and discrimination [1]. Malaysia has one of the fastest-growing HIV epidemics among MSM in Southeast Asia [2]. Surveillance data in 2019 suggest that over 1 in 5 (21.6%) MSM in Malaysia live with HIV [3]. HIV testing guidelines recommend high-risk MSM to be tested every 3 to 6 months [4,5]. However, MSM do not get tested optimally, with only 70.3% having ever been tested, 40.9% having been tested in the past year, and only 9.5% being tested more than once annually [6,7]. Moreover, in 2019, only 3% of MSM in Malaysia had adequate knowledge of HIV prevention, and only 36.7% reported ever having received HIV prevention services [3]. The low testing rates and inadequate prevention efforts are associated with high levels of stigma and discrimination against MSM, which are perpetuated in Malaysia, where same-sex sexual behaviors are criminalized by both secular and Shariah law [8]. To access HIV testing and prevention services, MSM in Malaysia must navigate individual and systemic barriers that perpetuate stigma and discrimination. Previous research has associated the heightened HIV burden among MSM in Malaysia with both etiological factors such as substance use during sexual encounters [9], which is linked to higher HIV transmission rates [10], and social factors such as perception of social stigma of pre-exposure prophylaxis use to prevent HIV [11], anxiety and fear for safety following the arrest of HIV prevention workers under legislation that codified sexual practices with persons of the same sex as criminal and unnatural [12], and health care workers’ discriminatory intentions against MSM [13]. Mobile health (mHealth) strategies that use theory-guided behavior change interventions and address both individual and systematic health care challenges have the potential to overcome multilevel barriers to HIV testing and prevention [14].

### mHealth and Chatbots

Mobile technologies’ increasing global penetration and capacity for swift information delivery and retrieval make mHealth a promising tool for HIV intervention, which requires timely monitoring of disease development at the population level as well as early detection, effective prevention, and efficient diagnosis at the individual level. mHealth strategies involve several modalities, including apps that are installed on smartphones or chatbots that are embedded within existing apps or websites. Multiple studies have identified mHealth as an effective means of facilitating HIV testing and prevention. For example, the HIVSmart app showed high efficacy in detecting new HIV infections and increasing self-test referrals [15]. A recent randomized controlled trial of an app intervention for increasing access to HIV testing and care among young cisgender men and transgender women highlighted the capacity of mHealth technologies for effective HIV prevention and real-time intervention delivery [16]. A meta-analysis of 41 studies evaluating 28 evidence-based mHealth interventions to support HIV self-management further revealed that mHealth interventions significantly improved individual-level medication adherence, mental health, and social support [17]. Despite the ample literature on the effectiveness of mHealth interventions, these studies have primarily involved apps, and chatbots are absent from HIV testing and prevention strategies.

Chatbots have the potential to create new opportunities to promote HIV testing and prevention. Chatbots are software-based programs that imitate human conversational agents to interact with users [18]. Users often converse with a chatbot on mobile platforms such as phones, laptops, websites, apps, and SMS text messaging, where the chatbot provides the information and solutions demanded by users through text responses or other engaging formats such as pictures, videos, and audio. Following the advances in natural language processing (NLP) and artificial intelligence (AI), AI chatbots, broadly referring to computer programs with minimal design interfaces embedded with AI to simulate conversation with human users, have been developed as mHealth strategies to support patient care and identified as an effective intervention for increasing health information delivery and promoting physical activity and a healthy diet [19]. A 2021 systematic review of the application of AI chatbots in health care and oncology accentuated the potential of integrating AI chatbots into clinical practice to reduce costs, improve patient outcomes, and enhance health practitioners’ work efficiencies [20]. Another recent review on the application of AI chatbots in digital mental health care suggested that, given AI chatbots’ unique ability to learn from and interface with patients, understanding the individual and contextual factors that might affect the impact of AI chatbots was particularly significant compared with conventional mHealth interventions [21]. Our study echoes this concern and aims to expand the understanding of AI chatbots’ health care application by demonstrating how individual and social factors are intricately linked to the mechanisms that affect people’s acceptance of AI chatbots as digital health interventions. Furthermore, in light of the onset of the COVID-19 pandemic, a recent review examined 61 chatbots used for pandemic public health response in 30 countries and highlighted chatbots’ “scalability, wide accessibility, ease of use, and fast information dissemination” as prominent features that served the interest of public health while calling for discussions on sophisticated chatbot design synergies [22]. In this study, we hope to deepen people’s understanding of AI chatbot design by illuminating user-centered features informed by multifaceted facilitators of and barriers to AI chatbot acceptance.

Although existing studies on the use of AI chatbots to improve health care outcomes encourage continuous investigation into the adoption of this technology, systematic discussions on contextual adaptions of AI chatbots to suit the unique dynamics of different populations, sociocultural contexts, and targeted health conditions remain scant. For example, a year-long prospective study of conversations between patients with breast cancer and an AI chatbot demonstrated the efficacy of using the chatbot to increase patients’ knowledge of breast cancer, medication adherence, and satisfaction with health care support [23]. Specifically, this study highlighted patients’ willingness to communicate sensitive and intimate information, such as their experiences of sexuality, with the AI chatbot, but whether patients’ perception of cancer-related social stigma existed or affected chatbot acceptance remained unexplored [23]. When adapting an AI chatbot to different health conditions and target populations in different sociocultural contexts, the same level of acceptance should not be assumed ubiquitously without deliberating the cultural and disease contexts in which an AI chatbot might receive sensitive personal information. Whether the same level of willingness can be found among stigmatized populations who are subjected to potential negative sociopolitical consequences because of disclosures of sensitive personal information, exemplified by the MSM population in Malaysia, remains to be investigated. Thus, this study heeds the significance of cross-cultural technology adaption and situates the examination of AI chatbot acceptance in the careful consideration of person-disease-context dynamics among MSM in Malaysia. Another study examined the acceptance of using a tuberculosis prevention AI chatbot among people who were vulnerable to tuberculosis in South Korea and found that the chatbot facilitated the dissemination of desired tuberculosis care information to at-risk populations [24]. Although the researchers acknowledged the stigmatization of tuberculosis in South Korea, the interrogation of health stigma remained outside the systematic consideration of how multilevel social influence could affect technology acceptance [24]. Therefore, we hope to incorporate systemic stigma into the discourse of social influence that affects technology acceptance, highlighting the interconnection among AI chatbot acceptance, individual needs, and macrosocial determinants of health.

### Purposes of the Study

To develop culturally tailored AI chatbots to promote HIV testing and prevention among MSM in Malaysia, researchers must identify the facilitators and barriers of chatbots among MSM and features that would promote acceptance. Such findings can play a critical role in informing and optimizing the design of the AI chatbot that is currently being developed, which may in turn influence its uptake and use [25]. Overall, this study aimed to increase our understanding of developing culturally tailored AI chatbots as mHealth interventions in a discriminatory environment against MSM and lay the groundwork for the future development, implementation, and scale-up of an MSM-friendly AI chatbot in Malaysia to improve HIV testing and use of prevention services. Specifically, we explored what influenced MSM’s acceptance of an AI chatbot designed to promote HIV testing and prevention using the unified theory of acceptance and use of technology (UTAUT).

### Conceptual Framework for Analysis

The UTAUT was used to guide the analysis of MSM’s acceptance of AI chatbots designed to promote HIV testing and prevention in Malaysia. The UTAUT is an extension of the technology acceptance model (TAM) that is commonly used to evaluate factors predicting people’s acceptance of new technologies. The TAM involves 2 constructs—perceived usefulness and perceived ease of use—as the primary predictors of users’ acceptance of new technologies [26]. The TAM was later expanded to the UTAUT, which includes (1) performance expectancy (perceived usefulness), (2) effort expectancy (perceived ease of use), (3) social influence, and (4) facilitating conditions as empirically proven predictors of technology acceptance and use intention [27].

The UTAUT was used as the analytical framework for 3 reasons. First, we hoped to use the insights learned from this study to guide the design of an HIV prevention AI chatbot. The UTAUT’s emphasis on users’ perception of what makes a technological tool useful rather than what is assumed to be useful by researchers and product designers would allow us to design the AI chatbot from a user-centered perspective. Second, it was important to use a validated framework such as the UTAUT, which has predictive value for identifying effective users’ acceptance of technology [28-33]. Third, although perceived self-efficacy, facilitating conditions, and system quality have been added to the UTAUT in evaluating health care technologies [34], there has been little investigation regarding the application of the UTAUT in HIV-specific health technology within cultural settings where stigma against HIV and MSM is pervasive. The standard definition of social influence in the UTAUT highlights an individual’s family’s, peers’, and friends’ influence on the individual’s technology acceptance and behavioral intention [27] rather than the influence of macro sociocultural determinants. Given the prevalent antihomosexuality sentiment and the heightened stigma toward HIV-infected MSM in Malaysia, we sought to provide insights into the adaptation of the UTAUT in assessing culturally specific and HIV-specific mHealth acceptance by incorporating stigma into the domain of social influence in the current model.

## Methods

### Eligibility Criteria

The inclusion criteria for the participants were as follows: (1) being cisgender men, (2) being aged ≥18 years, self-reporting (3) condomless sex with another man in the past 6 months and (4) HIV-negative or unknown status, and (5) speaking Bahasa Malaysia or English.

### Recruitment

**Figure 1:**
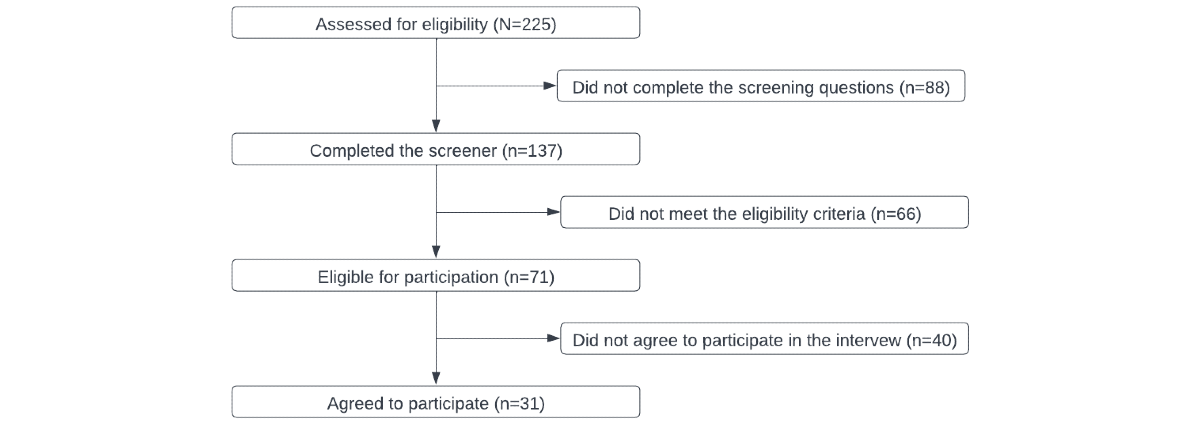
The screening process for research participants.

Figure 1 depicts findings from the recruitment strategy. The study setting was Malaysia, and the target population was MSM. A web-based screener was posted on MSM social networking apps, including Grindr, Hornet, and Blued, where MSM often meet to find sexual partners. Of the 225 people who initiated the web-based screener, 137 (60.9%) completed it, of whom 71 (51.8%) were eligible for participation. Of these 71 MSM, 31 (44%) agreed to participate in the focus group interviews and were enrolled. As part of the screening process, each participant was asked to complete a brief survey that included questions on demographic characteristics; HIV prevention practices; and self-reported measures of substance use, depression, and social media use.

### Procedures for the Focus Group Interviews

After obtaining informed consent, eligible participants were assigned to different groups based on their available times. The focus group interviews were conducted using Zoom (Zoom Video Communications). To ensure anonymity, the participants were instructed to set their preferences for their name (pseudonym) and video use. An experienced qualitative interviewer (JAW) with expertise in HIV prevention with MSM in Malaysia led all the interviews with other members of the research team to observe and record them. A total of 5 focus group interviews (range 5-9 participants each) were conducted using a semistructured interview guide (Multimedia Appendix 1) between July 2021 and September 2021. The interview guide was developed by adapting the evidence-based 5-phase process by Kallio et al [35]. First, we identified the prerequisite for using focus group discussions by assigning user technology acceptance and perception as the predetermined thematic concerns of our interviews. Second, informed by the purpose of formulative evaluation and previous literature, we decided on our 3 main topics for discussion. *Topic 1: attitude toward HIV testing* aimed to generate insight into the health behavior change targeted by chatbot interventions. *Topic 2: chatbot* focused on participants’ perceptions of the technology of interest. *Topic 3: social networking app* hoped to elicit user-centered suggestions regarding the platforms that should host MSM-friendly HIV prevention chatbots in response to the increasing mobilization of social media in the digital health industry [36]. Third, we formulated the interview guide by ensuring that the 3 topics proceeded logically from general to more specific questions, leaving room for interviewers to probe unanticipated but relevant issues raised during the discussion [37]. Fourth, we pilot-tested the guide among research team members not involved in the development of the interview guide. Finally, we presented the complete guide to the entire research team for approval.

During the focus group discussion, participants first watched an introductory video (90 seconds) about AI chatbots and how they worked, followed by open-ended questions regarding the facilitators and barriers associated with using an AI chatbot to promote HIV testing and prevention. Sample questions included “What do you think would be helpful for AI-chatbots to promote HIV testing?” and “Where would you expect to find an AI-chatbot designed to promote HIV testing and prevention?” Themes identified during the interviews were probed for further details as they emerged. Each group interview lasted 85 minutes on average (SD 5; range 81-94 minutes).

### Analysis

**Figure 2:**
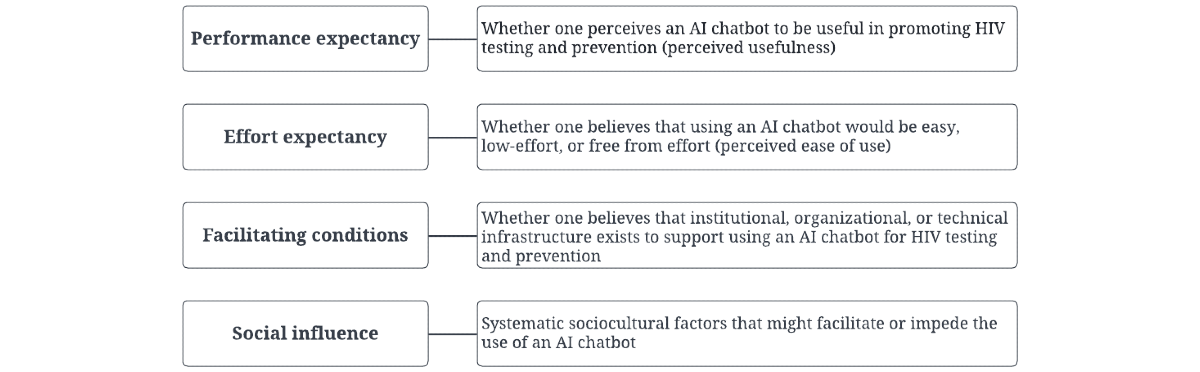
Summary of contextualized definitions of key terms. AI: artificial intelligence.

Descriptive statistics were calculated using SAS (version 9.4; SAS Institute) to summarize participants’ demographic characteristics; HIV prevention practices; and self-reported measures of substance use, depression, and social media use characteristics. The interviews were recorded, transcribed verbatim, and analyzed using thematic analysis. Emerging themes were mapped onto the 4 constructs of the UTAUT. Thematic coding was conducted using NVivo (version 9; QSR International) by 4 researchers (ZN, MAAH, WMI, and VT) who independently completed the initial round of coding. Each coder identified the patterns and themes that emerged from the findings regarding the benefits, concerns, and desirable features associated with using a chatbot for HIV testing and prevention. All coders collaboratively discussed their findings, resolved any discrepancies, and achieved saturation when no new themes were identified. The first author (MP) reviewed all the findings; identified similarities, differences, and redundancies among each coder’s findings; and organized the themes and illustrative quotations according to the four constructs of the UTAUT (Figure 2): (1) performance expectancy (ie, perceived usefulness), (2) effort expectancy (ie, perceived ease of use), (3) facilitating conditions, and (4) social influence. Later, a retrospective assessment of thematic saturation was conducted by the 4 coders by comparing the study findings with the themes in the System Usability Scale, a simple, 10-item attitude Likert scale that provides a global view of subjective assessments of usability [17]. We compared the findings with the System Usability Scale as it is a reliable and validated tool evaluating a wide range of technical themes, including a new technology’s use frequency, complexity, technical issues, integration of various functions, and inconsistency. Any new themes appearing in the System Usability Scale were double-checked by the 4 coders in the transcripts to ensure that our results were comprehensive enough to guide a wide variety of mHealth interventions and products. To increase reliability, themes were reviewed after coding, where the final coded themes were returned to 6 interviewees for accuracy check and to identify if there were any discrepancies between our interpretations and their intended opinions. All participants concurred with our findings.

### Ethics Approval

This study was approved by the institutional review boards of Yale University (ID 2000025910) and University of Malaya (ID 202049-8488). Participants reviewed all study-related risks and benefits and provided signed informed consent before participation. All procedures performed involving human participants were in accordance with the ethical standards of the institutional or national research committee and with the 1964 Helsinki declaration and its later amendments or comparable ethical standards. Participants were paid 45 Malaysian ringgits (US $10) after completing the study.

## Results

### Overview

Participants’ demographic characteristics are presented in Table 1. The mean age of the participants was 30.6 (SD 6.4) years, and they encompassed 4 ethnic groups: Malay (12/31, 39%), Chinese (16/31, 52%), Indian (1/31, 3%), and mixed (2/31, 6%). Among all participants, 48% (15/31) had never taken pre-exposure prophylaxis; 23% (7/31) had not been tested for HIV in the past 6 months; and 32% (10/31) had a PHQ-2 score >3, indicating a likelihood of depressive disorder.

**Figure 3:**
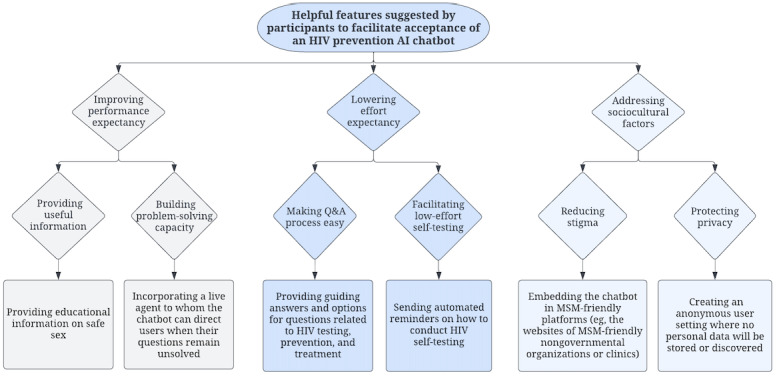
Helpful features for an HIV prevention artificial intelligence (AI) chatbot suggested by the participants. MSM: men who have sex with men; Q&A: question and answer.

Overall, most participants responded positively to using an AI chatbot for HIV testing and prevention. Multimedia Appendix 2 summarizes the key themes identified according to the 4 domains of the UTAUT with illustrative quotations. Figure 3 presents MSM’s preferred features of the AI chatbot suggested by the participants.

**Table 1:** Participant characteristics (N=31).

Variable			Values
Age (years), mean (SD)			30.6 (6.4)
**Ethnicity, n (%)**			
	Malay	12 (39)	
	Chinese	16 (52)	
	Indian	1 (3)	
	Mixed	2 (6)	
**Had been tested for HIV, n (%)**			
	In the last 3 months	12 (39)	
	In the last 4 to 6 months	12 (39)	
	In the last 7 to 9 months	2 (6)	
	In the last 10 to 12 months	5 (16)	
**Had ever taken PrEP^a^, n (%)**			
	Yes	16 (52)	
	No	15 (48)	
**Taking PrEP (n=16), n (%)**			
	Yes	11 (69)	
	No	5 (31)	
**Any drug use (past 6 months), n (%)**			
	Yes	7 (23)	
	No	24 (77)	
**Substance used (past 6 months), n (%)^b^**			
	Alcohol	11 (35)	
	Crystal methamphetamine	4 (13)	
	Gamma hydroxybutyrate	2 (6)	
	Cigarettes	5 (16)	
	Other drugs	1 (3)	
**PHQ-2^c^ score^d^, n (%)**			
	<3	21 (68)	
	≥3	10 (32)	
**Smartphone system, n (%)**			
	Android	15 (48)	
	iOS	16 (52)	
**App used, n (%)^e^**			
	WhatsApp	29 (94)	
	Facebook	25 (81)	
	Instagram	27 (87)	
	Telegram	19 (61)	
	Grindr	15 (48)	
	Twitter	24 (77)	
	Blued	6 (19)	
	WeChat	8 (26)	
	Discord	6 (19)	
	Jack’d	2 (6)	
	Scruff	2 (6)	

^a^PrEP: pre-exposure prophylaxis.

^b^Some participants used multiple drugs.

^c^PHQ-2: Patient Health Questionnaire-2.

^d^The PHQ-2 was used to screen for depression. Major depression was likely when the score was ≥3.

^e^Some participants used multiple apps.

### Performance Expectancy

#### Overall Perception

Participants reported both positive and negative perceptions regarding AI chatbots’ performance expectancy. They indicated that an AI chatbot’s ability to disseminate valuable information, solve routine questions, and raise HIV prevention awareness positively affected whether they perceived the AI chatbot to be useful. They also described concerns with information accuracy and skepticism of AI chatbots’ ability to provide emotional support and solve complex problems as the primary factors undermining performance expectancy.

#### Contributors to Positive Performance Expectancy

An AI chatbot’s capacity for information dissemination was a major contributor to positive performance expectancy. In total, 13% (4/31) of the participants explained that they perceived AI chatbots as useful “one-stop centers” for information with a simple user interface. A participant further elaborated on the relative advantage of using an AI chatbot to obtain information on HIV testing and prevention compared with canvassing multiple available sources and web-based platforms, which he described as a major obstacle to “getting tested often.” Another participant highlighted his perception of using an AI chatbot as a reliable source of information, stating that, compared with humans who might conceal information or twist facts, he perceived the information provided by AI chatbots as “factual” and more credible. Information regarding HIV awareness was also discussed. “Chatbot will be good for awareness purposes,” as one participant stated regarding AI chatbots’ usefulness in raising awareness for HIV testing and prevention. Nonetheless, another participant voiced skepticism, questioning that “how can chat robot send [relevant information to increase] awareness to the user?” In total, 13% (4/31) of the participants described that an AI chatbot was useful for providing solutions to users’ routine questions regarding HIV. A participant emphasized that “[if] it’s just a simple question, I believe the chatbot can help.” For instance, another participant stated the following:

...some people will ask simple questions like “where could I get tested” and the chatbot can totally help that by giving template answers.

#### Major Concerns

Despite some participants favoring AI chatbots as credible sources of information, a perceived lack of information accuracy and trustworthiness was reported by some as the primary reason that would hinder them from using AI chatbots. Specifically, with regard to obtaining information on HIV self-testing kits from AI chatbots, a participant commented the following:

I don’t know if I can trust those...products are working or not.

An overwhelming concern regarding AI chatbots’ ability to provide emotional support and a personal touch to health care was reported by 19% (6/31) of the participants. As participants stated, because of chatbots’ robotic nature, they “are not human alike,” “will reply [to the user] in really regulated answers,” and “there’s no replacing the human element, especially for those first-timers [in HIV-testing].” A total of 10% (3/31) of the participants emphasized the importance of human touch, reiterating their preference for in-person health care services. For example, regarding the preference for HIV service modality, a participant commented the following:

...at the end of the day, I think that most of them...wanted to reach out to talk to someone in person.

The same sentiment was echoed by another participant, who highlighted the following:

...sometimes you need another person in the same room offering you comfort and additional advice and support on what can be done and what needs to be done.

Participants’ perception of an AI chatbot’s ability to solve complex health problems was another major source of negative performance expectancy. A total of 19% (6/31) of the participants perceived using a chatbot as sometimes frustrating and limited in providing useful solutions to users’ complex questions and concerns. A participant elaborated that, in their previous experience using a chatbot, “it keeps being repetitive about the same kind of solution that I don’t need*.*” Another participant echoed the concern, stating the following:

[Chatbots] always go back to the same question that cannot get my question answered so sometimes it’s very frustrating.

Some participants also highlighted their desire to interact with a real person. As a participant claimed, “if it’s [the question] too complex, then I will prefer a human interaction.” Among the concerned participants, one nevertheless mentioned that, for complicated questions, a chatbot could be useful in “filtering and narrowing down those inquiries.”

#### Relevant Features Suggested by Participants

In total, 10% (3/31) of the participants further discussed the features that they perceived to be critical in improving an AI chatbot’s performance and usefulness. A participant suggested that the AI chatbot should provide educational information on safe sex, such as information on “*chemsex*,” defined as “the use of mephedrone, crystal methamphetamine and gamma hydroxybutyrate/butryolactone (GHB/GBL) to enable, enhance and prolong sexual interactions” [38], and “what to expect when they’re going to try [chemsex].” Another participant suggested having real-time human representatives to whom the chatbot could direct users when their questions could not be adequately answered by the chatbot. He elaborated the following:

...the chatbot cannot cater for every query or question that asked by the human, so I think it’s good to have a counselor as to fulfill the other questions that’s not being listed in the chatbot.

To increase reliability, the final coded themes were returned to 19% (6/31) of the interviewees for accuracy check. All participants agreed on our findings, and 33% (2/6) provided valuable feedback worthy of mention. They pointed out that AI chatbots needed to be able to provide information in a neutral tone and should not strive to be or pretend to be humans, although being able to converse smoothly was important.

### Effort Expectancy

#### Overall Perception

The participants reported both positive and negative perceptions regarding effort expectancy. The predominantly positive effort expectancy was associated with the convenience of using a chatbot. Major concerns included costs, technical difficulties, and the level of technical literacy required when using a chatbot.

#### Positive Contributors to Low Effort Expectancy

Many participants expressed favorable attitudes toward the convenience and ease of using a chatbot. A participant stated the following:

...in terms of convenience, I think it [an AI-chatbot] is brilliant, especially if you are planning to, you know, introduce it to the MSM community.

The participants perceived AI chatbots as convenient tools, specifically for their 24/7 availability and their ability to provide immediate responses. Compared with human agents, from whom one “might have to wait for a reply,” participants perceived an AI chatbot to be a more readily available source of help. A participant described the following:

I think the reason why I said [it is] convenient [is] because usually with chatbots you get your answer almost immediately.

Similarly, another participant elaborated the following:

...it’s just great because this hunting thoughts of whether you need to be checked up or not tends to come late at night when no personnel are actually available for you to speak to.

Overall, low effort and ease of use were reported as the major contributors to positive effort expectancy and, consequently, the facilitators of participants’ acceptance of AI chatbots designed to promote HIV testing and prevention.

#### Major Concerns

Positive perceptions aside, some participants spoke about the potential cost, technical difficulties, and required technical literacy when using AI chatbots as sources of negative effort expectancy. A participant speculated that using an AI chatbot would be costly, maintaining that “I think it’s going to be expensive, and I do not think that we should invest on it*.*” Another perceived obstacle to AI chatbots’ ease of use was the risk of technical breakdowns. In total, 6% (2/31) of the participants reported the same concern, one stating the following:

...sometimes it will break down. Then you have to do it again and again and again and again, and will be frustrated.

The other participant expressed similar frustration with the following:

...[imagine] your first attempt to just get HIV treatment...or PrEP...then suddenly a technical issue arises.

The participants generally perceived AI chatbots as high-technology products, prompting 6% (2/31) of the participants to express concerns regarding the difficulty of navigating an AI chatbot for the older population. A participant conjectured that more effort would be required from older users who might not be technologically savvy, stating the following:

...the younger generation definitely will find it easier to adapt...and as for the older generation...they still need to have someone to consult.

The other participant raised the same concern, reiterating the potential difficulty to use an AI chatbot for HIV testing and prevention among the older generation when “things start too complicated*.*”

#### Relevant Features Suggested by Participants

In terms of helpful features that could reduce use effort, some participants suggested that an AI chatbot should be designed to generate fast and seamless responses with guiding questions and options. A participant also envisioned that automatic information reminders on how to conduct HIV self-testing and notifications on progress throughout the testing process could enhance the AI chatbot’s ease of use. For example, he stated the following:

...you just need to enter something, [the chatbot will] give you the information and [to] book your parcel and to collect your parcel from the office.

#### Facilitating Conditions

Themes regarding both external and internal facilitating conditions emerged from participants’ responses. External facilitating conditions pertained to the overall HIV virtual care infrastructure, whereas internal facilitating conditions included the users’ attitudes toward HIV self-testing. Efficient linkage between users and health care professionals was a major external facilitating condition associated with the participants’ acceptance of the AI chatbot. If an individual believed that there would be an integrated HIV telemedicine infrastructure and an effective channel of information relay established between the AI chatbot and health care services, they would be more inclined to use the chatbot. A participant added that the automated linkage to health care and physician referral from an AI chatbot would encourage chatbot use because the referral process would make them feel more comfortable compared with going straight to a clinical setting as the chatbot would have mentally prepared them to consult health care professionals. They stated the following:

I would feel much more comfortable because the barrier is already broken when I checked with a chatbot, then I already let my guard down, then when it comes to the real person that can consult, then you feel much more open.

Participants’ attitudes toward self-testing were an important internal facilitating condition. On the one hand, most participants claimed that they would be encouraged to use an AI chatbot that could link them to HIV self-testing kits as they viewed HIV self-testing kits as a proprivacy, quick, and convenient testing method to learn their HIV status. By contrast, lack of self-testing awareness would undermine the incentives to use such a chatbot. As a participant reported, “we know that not everyone is aware of the self-testing kit,” and some participants “personally never heard of a self-testing kit*.*” Moreover, potential users’ concerns about the accuracy and trustworthiness of HIV self-testing results could hamper the use of the AI chatbot to obtain HIV self-tests. As emphasized by a participant, he “would like to be assured by a professional that my test [result] is right.”

### Social Influence

#### Overview

Our participants also reported sociocultural factors that would facilitate or impede their acceptance of an AI chatbot, including stigma, fear of discrimination, privacy concerns, and drug-related legal concerns. Many participants mentioned the discriminatory cultural climate against MSM and the HIV stigmatization targeted at MSM in Malaysia:

HIV is still a taboo [in Malaysia]

...there is huge stigma on the subject. People don’t generally engage in the conversation about HIV.

...there are some judgments during those [HIV] checkups, and it deters me from any future visits.

As a result, participants perceived an AI chatbot as a helpful learning and service tool in an environment with discrimination and stigmatization, believing that self-testing facilitated by the AI chatbot could help MSM avoid stigma and discrimination and, thus, increase HIV testing rates.

On the issue of avoiding discrimination and stigmatization, 10% (3/31) of the participants discussed the issue of HIV testing privacy, emphasizing that an AI chatbot could be a helpful means to protect their identity and privacy compared with routine clinical testing, especially for people “not willing to be tested by professionals.” A participant elaborated the following:

...once told the nurse, the nurse told another nurse, and another nurse, and [eventually] everyone knew [I am] there for HIV testing.

The benefit of using an AI chatbot for HIV self-testing in terms of safeguarding MSM’s privacy was reiterated by another participant, who reported that he had to commute to another district far away from his current living address for HIV testing to protect his privacy. He stated the following:

I think a chatbot is very helpful because when I took my HIV test, I was not checking at my area. I live at Putrajaya, but I checked at Johor because I need to protect my privacy.

Some participants further reported that, coupled with the fear of social stigma against HIV and MSM in Malaysia, they were concerned about the criminalization of substance use. As explained by a participant, they would be concerned about information censorship and unintentional engagement with illegal drug use if an AI chatbot was perceived as providing information on HIV-related or MSM sex-related drug use, which could in turn hamper their acceptance of the AI chatbot. Another participant further elaborated the following:

I just feel that by having so much you know, putting up so much information out there, via chatbot I’m afraid that you know it seems that we are promoting of using drugs but, so we have to be quite careful with the information that we display out.

#### Relevant Features Suggested by Participants

Owing to the preponderant stigma associated with same-sex sexual behaviors and HIV in Malaysia, most MSM participants preferred having an AI chatbot embedded in MSM-friendly nongovernmental organizations’ or clinics’ websites. Participants believed that these platforms may offer greater privacy and a more secure virtual environment than a social networking app such as Facebook, Twitter, or Instagram, where users would be required to register using their personal information. Some participants also indicated that using MSM-friendly platforms could facilitate more effective offline linkage to HIV testing and prevention services as they would feel less weary about reaching out to health workers affiliated with MSM-friendly institutions. A participant further emphasized the importance of having an anonymous user setting, reiterating privacy concerns and stating that “people might not have the comfort to go to a person and let them know they want to do the testing*.*”

## Discussion

### Principal Findings

In settings such as Malaysia, where same-sex sexual behaviors are illegal in secular and Shariah law, there are several key design and implementation factors that must be considered to improve the acceptability of AI chatbots that target HIV prevention in MSM. The UTAUT framework is an ideal heuristic for examining barriers and facilitators for new technologies; therefore, we analyzed the potential facilitators and barriers associated with MSM’s acceptance of AI chatbots aimed at promoting HIV testing and prevention based on the UTAUT, categorizing participants’ insights into performance expectancy, effort expectancy, facilitating conditions, and social influence. There were no additional or anomalous themes that stood incompatible or incongruent with the 4 aforementioned factors. The findings suggested that, with the right design, features, and platforms, the implementation of an AI chatbot to promote HIV testing and prevention could be acceptable to MSM, an at-risk population highly vulnerable to HIV infection in Malaysia.

The UTAUT states that positively perceived performance expectancy and perceived low effort to use facilitate use intention and technology acceptance [27]. On the basis of our results, an AI chatbot’s perceived usefulness and ease of use stem from its ability to disseminate valuable HIV-related information, provide easy answers to users’ questions, and raise HIV prevention awareness, and from its convenience and 24/7 availability. The participants placed a high value on an AI chatbot’s ability to operationalize these features. In light of participants’ opinions and direct feedback on preferred features, the design of an HIV prevention AI chatbot needs to (1) incorporate security and privacy settings and ensure anonymity to protect chatbot users; (2) have easily reachable human agents and a customer support section embedded in the AI chatbot interface; (3) have a learning center or information center embedded in the interface and consider a wide variety of educational, awareness-raising, and informative materials to be incorporated into the information center; (4) undergo meticulous model training to answer complex questions with high accuracy and clarity; (5) provide signposting questions to break down complex questions to facilitate complex problem-solving; (6) have a user-friendly and intuitive interface that does not undermine older users’ ability to navigate the AI chatbot; (7) provide a navigation manual or chatbot function tour for newly registered users; and (8) have an efficient and responsive technical support sector where technical issues can be reported and investigated 24/7. As demonstrated in previous applications of the UTAUT in assessing technology acceptance, performance expectancy and effort expectancy had a significant impact on use intention, although performance expectancy is often reported as a stronger factor [39-43]. Coupled with these findings, the qualitative insights obtained from this study further demonstrate the necessity to ensure and continuously enhance an HIV prevention AI chatbot’s user-centered designs grounded on users’ feedback on the chatbot’s performance and ease of use.

Findings from this study further expand the facilitating conditions that may encourage HIV prevention AI chatbot acceptance and use. Incorporating linkage to health care professionals and services should be considered in the design and implementation of the chatbot. Not only should an HIV prevention AI chatbot be designed as a one-stop center for information provision, but it should also be envisioned as a hub of health care connection that provides effective linkage and communication between health care consumers and health care providers. This finding accentuates the importance of socioecological and systematic thinking in the design of digital health tools. Health care services should ideally be treated as an ecosystem rather than as separate localities of services where one service locale readily affects the acceptance and use of another. Eliciting participation from local HIV clinics, HIV testing sites, and HIV health care providers; incorporating them as part of an HIV prevention AI chatbot’s user-chatbot-provider ecosystem; and allowing the AI chatbot to readily transfer patient data upon request to health care services could prove crucial in improving the implementation outcomes of the chatbot, such as acceptability and sustainability.

It should also be noted that individual internal facilitating conditions, such as attitudes toward HIV self-testing, are intricately shaped by external infrastructural conditions. If an AI chatbot intends to encourage HIV self-testing by linking users to HIV home testing services, the overall functionality of the self-testing service system and the information dissemination and educational infrastructure placed by HIV home test vendors would be a significant factor in shaping participants’ attitudes toward HIV self-testing and, consequently, their attitudes toward the acceptance of the HIV prevention chatbot. Therefore, the chatbot development team could reach out to HIV home test vendors to encourage educational campaigns regarding their products, with an emphasis on the efficacy, transparency, and limitations of their HIV home testing kits. To help shape users’ positive perception of self-testing, the AI chatbot could also incorporate a function that allows users to compare the quality of different self-testing kits, such as their price, sensitivity and specificity, positive and negative predictive values, and user reviews and satisfaction ratings. As Venkatesh et al [27] maintained, “facilitating conditions have a direct positive effect on intention to use.” As supported by a previous mixed methods study on the acceptance of an informational antituberculosis chatbot among South Korean adults, facilitating conditions, specifically “the extent to which users think organizational and technical infrastructure exists to support the use of antituberculosis chatbots,” showed a strong connection with the acceptance of the chatbot by patients with tuberculosis [24]. Given the findings of this study and the previous literature, the design of an HIV prevention AI chatbot should adopt systematic thinking by creating an efficacious user-centered chatbot HIV care service system and investing in improving facilitating conditions beyond focusing on the characteristics of the chatbot itself.

This study also provides insights into how social influence might affect the acceptance of an HIV prevention AI chatbot. Social influence is originally defined by Venkatesh et al [27] as “the degree to which an individual perceives that important others believe he or she should use the new system” in the UTAUT. The implication of this factor is that people’s behavior is affected by and adjusted to others’ perceptions of them. In this study, the definition of social influence goes beyond how one’s behavioral intention is directly affected by important others’ perceptions of them and focuses on how sociocultural climate and collective social perceptions, such as stigma, discrimination, and criminalization, affect individuals’ behavior. The interviews revealed that the anti–lesbian, gay, bisexual, transgender, and queer culture and the taboo around HIV in Malaysia shape MSM’s privacy concerns around HIV care and act as an impetus for MSM to resort to proprivacy technologies such as an HIV prevention AI chatbot, which would allow them to bypass human interaction in HIV testing and care services. Although previous studies attempting to empirically validate the UTAUT demonstrate that social influences have an inconsistent effect on technology acceptance across different settings [26], this study points to a new lens through which social influence can be examined. Instead of focusing purely on how individual behavior is affected by important others’ perceptions of them, macrolevel social influences, such as cultural stigma, discrimination, social inequality, health justice, and identity oppression, can be further investigated as potential variables of social influence and, thus, technology acceptance. The consideration of these systematic sociocultural factors could prove particularly salient for the design of mHealth interventions targeted at systematically disadvantaged at-risk populations who experience a high burden of social marginalization and health care inequality.

We offer 2 major suggestions for researchers worldwide when designing culturally tailored AI chatbots. First, the examination of the social determinants of the health outcome or disease that an AI chatbot aims to address should be an integral part of the design process. The understanding of the sociocultural mechanism that drives a specific population’s behaviors and attitudes toward a health condition and health care can be used to decide what health care stakeholders an AI chatbot should aspire to connect to effectively encourage health-promoting actions among targeted populations. Different levels of stakeholders that can be connected to form a user-centered AI health care ecosystem include users, families, friends, health care providers, community health workers, nonprofit organizations, governmental agencies, policy makers, researchers, and community support networks. Second, although upstream social determinants such as health policies, discrimination and sociocultural stigma, and systematic health care inequality cannot be single-handedly solved by one mHealth technology, features such as empathetic NLP and human custom support agent empathy training that could assuage users’ system-, stigma-, or discrimination-induced anxiety around a health condition can be incorporated into an AI chatbot to encourage positive attitudes toward health and care seeking. The latest publication by Rahmanti et al [44] on a chatbot designed with artificial empathy features for weight management demonstrated the promising impact of empathetic NLP on engendering long-term behavior change and fostering emotional and social support. Thus, we recommend the incorporation of artificial empathy into the design of AI chatbots that target stigma-ridden and anxiety-inducing health conditions in high-risk populations. Efforts should also be dedicated to training culturally and socially tailored empathetic responses in AI chatbot NLPs rather than assuming one-response-fits-all empathetic languages. Empathy training for health care workers has been described as of “unquestionable importance” [45]. In the virtual space, an AI chatbot’s human support agents undertake the same tasks of user or patient communication and problem-solving as health care workers in the clinical setting. The final implementation and operationalization of the entire AI chatbot network, where the chatbot may refer users to human agents for further support, necessitates the consideration of empathy training not only within the chatbot programming but also within the human extension of the chatbot system. Anti-implicit bias training and compassionate care may be considered as part of the human support empathy training.

Finally, the UTAUT states that the effect of performance expectancy, effort expectancy, facilitating conditions, and social influence is “moderated by age, gender, experience and voluntariness of use” [27]. Although this study did not systematically explore the moderating effects of these factors, it indicated the potential disparity in the acceptance of an HIV prevention AI chatbot between younger and older users. The older population may expect a higher level of difficulty in using the chatbot because of limited technological literacy. Future randomized controlled trials that examine the moderating effects of multi-sectional identities such as age, experience, socioeconomic background, and education level could prove valuable in demonstrating whether, given the same AI chatbot, use intention and acceptance of technology would differ among different populations.

Acceptability is a widely assessed implementation outcome, defined as the perception among implementation stakeholders that a given treatment, service, practice, or innovation is agreeable or satisfactory [46]. Both *acceptance* and *acceptability* were mentioned in this study, but the distinction between the 2 terms should be noted. We used *acceptance* as intended in the UTAUT, denoting users’ intentions to use a technology [27]. This study specifically investigated the potential barriers and facilitators associated with users’ intention to use AI chatbots for HIV testing and prevention. We used *acceptability* as intended in implementation science, denoting an implementation outcome [46]. Insights into what affects users’ acceptance were used to discuss the implications of this study on the implementation outcome of the chatbot.

Overall, the study’s findings illustrate the potential facilitators of and barriers to MSM’s acceptance of an AI chatbot as a means to promote HIV testing and prevention, which are classified according to the 4 constructs of the UTAUT. Insights related to performance expectancy, effort expectancy, facilitating conditions, and social influences all suggest that an HIV prevention AI chatbot could have high acceptance among MSM in Malaysia. Our evidence speaks to the imperative of using user-centered design to enhance AI chatbots’ convenience, availability, problem-solving capacity, and ability to provide valuable information and raise HIV prevention awareness. Conversely, negligence of these factors could impede user acceptance of the chatbot. Our findings also point to a greater chance of user acceptance if systematic and socioecological thinking is effectively incorporated into the design of AI chatbots, where linkage to health care services and vendor-consumer information dissemination are well supported by the functions and health care network incorporated into the chatbot. This study further provides hypotheses regarding the impact of health stigma and discrimination on HIV-related health technologies, which warrants further investigation and validation through randomized controlled trials and quantitative regression analysis. As the preimplementation formative research for an HIV prevention AI chatbot in Malaysia evolves, these insights will be used to inform the design of the AI chatbot and the testing, evaluation, and optimization of the implementation outcomes of the AI chatbot, such as appropriateness, feasibility, and sustainability, in the mid- and postimplementation stages of the AI chatbot development program.

### Limitations

Although this study contributes important knowledge to the understanding of the use of AI chatbots as an HIV prevention strategy, the following limitations must be acknowledged. First, given the preimplementation phase and the explorative nature of this study, the findings neither statistically tested any hypothesis regarding user acceptance of AI chatbot technology nor confirmed the actual acceptability of an AI chatbot. Further quantitative or mixed methods research should be conducted to corroborate whether the reported factors are related to the actual acceptability and usability of an MSM-targeted AI chatbot in Malaysia. Second, all interviews were conducted in English, which may have limited the interpretation of the data. A large sample stratified by English and non-English speakers may reveal other facilitators and barriers associated with participants’ acceptance of an AI chatbot. Nevertheless, as the aim of this study was not to test a hypothesis but rather to generate in-depth insights, this sample would not undermine the significance or validity of the participants’ opinions [47]. Third, convenience sampling was used in the recruitment of interviewees. Although the recruited participants represented a wide range of demographic characteristics (shown in Table 1), thus mitigating the risk of overly narrow insights because of homogeneous population characteristics, random sampling could strengthen the study by incorporating a greater demographic diversity of participants, thus generating a greater breadth of insights. Furthermore, given the taboo culture around HIV and nonheterosexual orientation in Malaysia, participants might have withheld information regarding sensitive personal experiences and social issues regarding HIV care, which might otherwise provide more insights for this study. We also acknowledge the possibility of the misreporting of MSM identity among participants, although we believe this was highly improbable as participants voluntarily responded to our recruitment on social media and, in a sociopolitical environment that discriminates against MSM, the motivation to take on a false marginalized identity would be irrational. In addition, this study only obtained potential MSM users’ perspectives. An investigation of health care providers’ and other stakeholders’ perspectives, such as community health workers and local policy stakeholders, could have provided a richer understanding of what might encourage or discourage the acceptance and use of an AI chatbot in Malaysia. Further research is warranted in this regard.

### Conclusions

In the absence of research that examines what influences the acceptance of AI chatbots as tools for HIV prevention in an anti–lesbian, gay, bisexual, transgender, and queer cultural setting, this study contributes to the understanding of what may affect users’ acceptance of AI chatbots designed to promote HIV testing and prevention in Malaysia. The results of this study present the perceived benefits and concerns of using an AI chatbot among MSM and apply the UTAUT framework to understand the acceptance of health technology used for the prevention of stigma-ridden diseases. Not only can these findings inform the future design of AI chatbots aimed at promoting HIV testing and prevention, but they also provide direction for the integration of health stigma into the UTAUT’s application in health technology settings.

## Appendix

Multimedia Appendix 1 Interview guide of the chatbot project—men who have sex with men.

Multimedia Appendix 2 Participants’ insights with illustrative quotes.

## Abbreviations

AI: artificial intelligence
mHealth: mobile health
MSM: men who have sex with men
NLP: natural language processing
TAM: technology acceptance model
UTAUT: unified theory of acceptance and use of technology
