# Supplementary material for: Formative Evaluation of the Acceptance of HIV Prevention Artificial Intelligence Chatbots By Men Who Have Sex With Men in Malaysia: Focus Group Study
Source: JMIR Form Res. 2022 Oct 6;6(10):e42055. doi: 10.2196/42055 (PMC9585446; doi:10.2196/42055)
Supplement: Multimedia Appendix 1 [file formative_v6i10e42055_app1.pdf]

Appendix 1. Interview Guide

Interview Guide of AI-Chabot Project - MSM

Introduction

Hello, everyone! Thank you very much for joining our focus group discussion about new strategies for improving HIV testing and HIV prevention in Malaysia. We are delighted to have your involvement in this project. In this focus group discussion, we will be looking for your input, thoughts, and opinions regarding an AI-chatbot that we are designing to improve access to HIV prevention services - such as HIV testing and pre-exposure prophylaxis (PrEP) – among gay men or men who have sex with men in Malaysia.

The goal of the AI-chatbot is to make it easier for men to access HIV testing and PrEP services through your mobile phone. Like how the Grab app made it easier to get a taxi, we want our chatbot to make it easier to get HIV testing and PrEP. We want the chatbot to be as user-friendly, convenient, and acceptable as possible. To accomplish this, however, we need your input and guidance to make sure we get it just right. We are grateful for your input and feedback, which will make an impact on the look, feel, and function of the AI-chatbot.

I'll be asking some questions and opening the discussion to the group. I am eager to hear from everyone and to learn from you and your experiences. My job will be to monitor the group, make sure we all have opportunities to share our experiences, and make sure we are respectful of one another. There are no correct or incorrect answers. Nor is it required that we achieve consensus. We just want to hear as truthfully as possible from you about the questions asked. In fact, the more opinions the better. We don't have to agree, but I do ask each of us to be open and respectful of each other.

Preamble: HIV testing

The World Health Organization recommends, for most gay men, to be tested for HIV twice a year, and for some gay men more frequently, every three months. To find out what gets in the way of gay men or men who have sex with men getting an HIV test done as recommended, our team has conducted a group interview among participants last month and found that gay men or men who have sex with men did not test optimally.

Question 1: Chatbot

Preamble: Before I ask you the question, I would like to define a few things to make sure we are all on the same page.

Have you used a chatbot before? Is there anyone who is not familiar with chatbot?

DEMONSTRATE A CHATBOT TO PARTICIPANTS
using the YouTube video (What are Chatbots?)
https://www.youtube.com/watch?v=pX6zqaEHAdw

Now, what I would like to do is to pose a question. After you think about it for a few minutes, I will start to make a list of what you think.

<u>What do you think would be helpful for artificial intelligence (AI) chatbots to do for HIV testing?</u>

Potential prompts:

i. Ordering HIVST kits, lubes and condoms
ii. Giving instruction on the testing process
iii. Finding closest clinics to get tested
iv. Responses in text, pictures, or voice?
v. Consulting with the doctor using the Chatbot
    1. Access lab results via the Chatbot
    2. Review lab results with the doctor via the Chatbot

<u>Question 2</u>: Social-networking app

Preamble: You have made a list of the functions that you want to see in the AI-chatbot (GO OVER THE TOP THREE RANKINGS). Next, we would like to discuss which social-networking apps you want to use to embed the chatbot for sexual health.

Now, like what we did earlier, what I would like to do is to pose a question and after you think about it for a few minutes, start to make a list of what you think.

<u>Where would you expect to find such an AI-chatbot designed to promote HIV testing and prevention?</u>

Potential prompts:

i. Which type of chatbot participants prefer? A chatbot within a website page or a chatbot within an app?
ii. Telegram?
