# Supplementary material for: Formative Evaluation of the Acceptance of HIV Prevention Artificial Intelligence Chatbots By Men Who Have Sex With Men in Malaysia: Focus Group Study
Source: JMIR Form Res. 2022 Oct 6;6(10):e42055. doi: 10.2196/42055 (PMC9585446; doi:10.2196/42055)
Supplement: Multimedia Appendix 2 [file formative_v6i10e42055_app2.pdf]

Appendix 2. Participants' Insights with Illustrative Quotes

Domain and themes	Quotations
**Domain 1: performance expectancy**	
Accuracy concerns	• Concerned about inaccurate and untrustworthy information provided by an AI[a] chatbot[b]<br>o "I don't know if I can trust those...products are working or not."
Concerns about emotional support	• Concerned about lack of personal touch and lack of emotional support because of chatbots' robotic nature[b]<br>o "Too robotic and not human alike."<br>o "Like the sort of like robotic reply that is sometimes implemented in certain cases where you know if you're asking a certain way, and then the chatbot will reply to it in a really regulated answers."<br>o "Sometimes you need another personnel in the same room offering you comfort and additional advice and support on what can be done and what needs to be done."<br>o "At the end of the day, I think that most of them whenever they wanted to reach out to talk to someone in person."<br>o "I prefer to ask something by calling, calling someone or face to face."<br>o "There's no replacing the human element, especially for those first-timers."
Information dissemination	• Perceived an HIV prevention AI chatbot as a helpful one-stop center for information[c]<br>o "I had to go to many different unofficial sources, just to get that basic information. I feel that we have many difficulties. No wonder that not many people get tested often."<br>o "...majority or important things I can get it from chatbot."<br>o "...if we talked to [a] person we don't have the fact about something and I think if the chatbot so far, so the information that I got from the chatbot will be like the fact."<br>o "I can simply just ask the chat bot, so all the information I can straight away get from there."
Problem-solving	• Perceived AI chatbots as useful tools for basic information acquisition[c]<br>o "I think chatbot will be good for them to get their basic knowledge of it."<br>o "It's just a simple a simple question that I believe the chat bot can help them."<br>o "I will receive a lot of questions and with the chat bot, would totally help...I've been getting like over 200 private messages, and you know...I'm working with a women's reproductive help, help, help line...people will ask, some people will ask simple questions like 'where can I get tested' and the chat bot can totally help that by giving template answers."

	• Perceived AI chatbots as helpful in filtering and breaking down questions[c]<br>o "For complicated issues yeah but I guess that's the brilliance of it isn't it? I mean chatbot would help to filter and narrow down those inquiries."<br>• Perceived AI chatbots as frustrating and limited in providing useful solutions to complex problems[b]<br>o "Recently, I think one of the internet service providers, had a chatbot as well, but I was required very specific technical details which was not able to give me and it keep being repetitive about the same kind of solution that I don't need."<br>o "Like some of the shopping platform they do have this chat Bot and the problem is that always go back to the same question that cannot be get my get my question answer so it's sometimes it's very frustrated."<br>o "Depends on the thing that I wanted to ask. If it's too complex, then I will prefer a human interaction."<br>o "The first thing that comes to my mind is I'm about to be disappointed."<br>o "I am actually from a marketing background. So, I use this chat bot a lot and sometimes it irritates me...each time I use, it doesn't mean that I'll get a direct answer from them."<br>o "When we enter any kind of question or any kind of inquiry, they don't catch, or they can they don't get what we tried to ask."
Raising awareness	• Perceived an HIV prevention AI chatbot as helpful in raising awareness of HIV prevention[c]<br>o "Chatbot will be good for awareness purposes."<br>• Perceived an AI chatbot as limited in raising awareness[b]<br>o "It [the chatbot] doesn't proactively reach out to you."<br>o "How can chat robot send awareness to the to the user of the chat box itself?"
Desirable features	• Educational information on safe sex could be helpful[c]<br>o "Another thing is for the user itself, maybe pre before they go into that activity, they can get some information on what to expect when they're going to try to have substance, you know let's say you're on Grindr let's have some fun and then he can ask like oh what kind of substance, you guys are using oh G-Water and ice so before he participates in that activity, you know, like maybe he's not sure if he's ready for that kind of experience that he can go to the chatbot."<br>• Having real-time human representatives to whom an AI chatbot can direct users would be helpful[c]<br>o "The chat bot cannot cater for every query or, or question that asked by the human, so I think it's good to have a counselor as

	to fulfill the other questions that's not being listed in the chat bot."
**Domain 2: effort expectancy**	
Convenience	• Perceived an AI chatbot as a convenient tool because of its fast response and 24/7 availability[c]<br>o "In terms of convenience, I think it's brilliant, especially if you are planning to, you know, introduce it to the MSM community."<br>o "I think the reason why I said convenient because usually with chat bots you get your answer almost immediately."<br>o "I think, maybe it's um you know if it's a human you might have to wait for a reply."<br>o "It is also very convenient and the fact that it's available 24/7. It's just great because this hunting thoughts of whether you need to be checked up or not tends to come late at night when no personnel are actually available for you to speak to."
Cost	• Perceived high costs could hamper acceptance of an AI chatbot[b]<br>o "I have to [say] sorry about like umm I know that the conditions has you know whether it's intricacies of complication and also the cost right I think it's going to be expensive, and I do not think that we should invest on it."
Technical errors	• Perceived risk of technical breakdowns could undermine acceptance of an AI chatbot[b]<br>o "Sometimes is that it will breakdown. Then you have to do it again and again and again and again, and will be frustrated."<br>o "[imagine] your first attempt to just get HIV treatment...or prep...Then suddenly [a technical issue arise]."
Technological literacy	• Technological literacy disparity could create uneven benefits between younger and older populations[b]<br>o "The younger generation definitely will find it easier to adapt...and as for the maybe the older generation...they still need to have really have someone to consult."<br>o "Let's say when things start too complicated...then there will be an issue for them [the older generation]."
Desirable features	• An AI chatbot that could generate fast and seamless responses with guiding questions and options would be helpful[c]<br>o "You just need to enter something, something [will] give you the information and [to] book your parcel and to collect your parcel from the office. So I think that's a great idea if we can use it when we asked about HIV."
**Domain 3: facilitating conditions**	
Linkage to health care professionals	• The ability to link users to health care professionals could encourage the use of AI chatbots[c]<br>o "...I would feel much more comfortable because the barrier is already break when I checked with a chatbot then I already let

	my guard down, then when it comes to the real person that can consult, then you feel much more open."
Attitudes toward HIV self-testing	• Lack of self-testing awareness could hamper the acceptance of an HIV prevention AI chatbot[b]<br>o "We know that not everyone is aware of the self-testing kit."<br>o "I personally never heard of a self-testing kit."<br>• Concerns about self-testings' accuracy and trustworthiness could hamper the use of AI chatbots[b]<br>o "I would like to be assured by a professional that my test [result] is right."<br>o "I don't know if I can trust those sellers if their products are working or not."
**Domain 4: cultural-political climate (expanded TAM[d] through findings from this research)**	
Stigmatization and privacy concerns	• Perceived an HIV prevention AI chatbot as a helpful learning and service tool for MSM[e] in an anti-LGBTQ[f] culture[c]<br>• Considered an HIV prevention AI chatbot as a tool for helping MSM avoid stigma and discrimination and, thus, increasing HIV testing rates[c]<br>o "HIV is still a taboo" (in Malaysia).<br>o "There is huge stigma on the subject. People don't generally engage in the conversation about HIV."<br>o "There are some judgments during those checkups, and it deters me from any future visits."<br>• Perceived chatbot-guided self-testing as a helpful means to avoid discrimination and protect personal privacy compared with clinical HIV testing[c]<br>o "Once told the nurse, the nurse told another nurse, and another nurse, and [finally] everyone knew [I am] there for HIV testing."<br>o "I think a chatbot is very helpful because when I took my HIV test, I was not checking at my area. I live at Putrajaya, but I checked at Johor because I need to protect my privacy."<br>o "I think it will be helpful to someone who is not willing to be tested by professional."
Legal concerns	• Fear of engaging with information censorship/illegal activities could hamper the acceptance of an HIV prevention AI chatbot[b]<br>o "I don't know I just feel that you know by having so much you know, putting up so much information out there, via chatbot I'm afraid that you know it seems that we are promoting of using drugs but, so we have to be quite careful with the information that we display out."
Desirable features	• Embedding an HIV prevention AI chatbot in an MSM-friendly platform, such as websites of MSM-friendly NGOs[g] or clinics[c]<br>• An anonymous user setting could be helpful[b]

	○ "Mostly, the way to know about the process now is, you have to engage the person, which some people might not have the comfort to go to a person and let them know they want to do the testing, maybe they just be for this anonymity."

[a]AI: artificial intelligence.
[b]Barriers to men who have sex with men's acceptance of AI chatbots designed to promote HIV testing and prevention.
[c]Facilitating conditions of men who have sex with men's acceptance of AI chatbots designed to promote HIV testing and prevention.
[d]TAM: technology acceptance model.
[e]MSM: men who have sex with men.
[f]LGBTQ: lesbian, gay, bisexual, transgender, and queer.
[g]NGO: nongovernmental organization.
